# Liposome-Mediated Cellular Delivery of Active gp91^phox^

**DOI:** 10.1371/journal.pone.0000856

**Published:** 2007-09-12

**Authors:** Bruno Marques, Lavinia Liguori, Marie-Hélène Paclet, Ana Villegas-Mendéz, Romy Rothe, Françoise Morel, Jean-Luc Lenormand

**Affiliations:** 1 HumProTher, UMR-CNRS 5525, Université Joseph Fourier, Centre Hospitalier Universitaire, Laboratoire d'Enzymologie/DBPC/BP 217, Centre Hospitalier Universitaire de Grenoble, Grenoble, France; 2 GREPI, TIMC-Imag, UMR-CNRS 5525, Université Joseph Fourier, Centre Hospitalier Universitaire, Laboratoire d'Enzymologie/DBPC/BP 217, Centre Hospitalier Universitaire de Grenoble, Grenoble, France; Ordway Research Institute, United States of America

## Abstract

**Background:**

Gp91^phox^ is a transmembrane protein and the catalytic core of the NADPH oxidase complex of neutrophils. Lack of this protein causes chronic granulomatous disease (CGD), a rare genetic disorder characterized by severe and recurrent infections due to the incapacity of phagocytes to kill microorganisms.

**Methodology:**

Here we optimize a prokaryotic cell-free expression system to produce integral mammalian membrane proteins.

**Conclusions:**

Using this system, we over-express truncated forms of the gp91^phox^ protein under soluble form in the presence of detergents or lipids resulting in active proteins with a “native-like” conformation. All the proteins exhibit diaphorase activity in the presence of cytosolic factors (p67^phox^, p47^phox^, p40^phox^ and Rac) and arachidonic acid. We also produce proteoliposomes containing gp91^phox^ protein and demonstrate that these proteins exhibit activities similar to their cellular counterpart. The proteoliposomes induce rapid cellular delivery and relocation of recombinant gp91^phox^ proteins to the plasma membrane. Our data support the concept of cell-free expression technology for producing recombinant proteoliposomes and their use for functional and structural studies or protein therapy by complementing deficient cells in gp91^phox^ protein.

## Introduction

Gp91^phox^ protein is the catalytic subunit of the NADPH oxidase complex in human neutrophils and is involved in the electron transfer from NADPH to molecular oxygen O_2_ [1]. Gp91^phox ^is a transmembrane glycoprotein which physiologically associates with the p22^phox^ subunit to form flavocytochrome *b_558_* [2]. MALDI and nanospray LC-MS/MS methods have delineated the transmembrane domains of gp91^phox^ [3]. This protein contains six putative α-helices and three extracellular loops in which the glycosylation sites (Asn132, Asn149 and Asn240) are comprised in loops 2 and 3 [4]. In its active state, NADPH oxidase is a multi-component enzyme complex composed of cytochrome *b_558_* (gp91^phox ^and p22^phox^) and cytosolic factors (p67^phox^, p47^phox^, p40^phox^, Rac and Rap1A) that translocate at the membrane surface from cytosol upon stimulation. From this transfer and through the assembly of the constituents, NADPH oxidase is activated [5]. Upon infection or stimulation with inflammatory mediators, NADPH oxidase from neutrophils generate O_2_^−^ and then oxygen derivatives, or ROS, which are necessary for the defense of the organism.

Recent studies on the C-terminal part (amino acids 221 to 570) of gp91^phox^ protein show that the cytoplasmic domain retains a high NADPH diaphorase activity [6] that can be stimulated in the presence of cytosolic proteins Rac and p67^phox^. This supports the hypothesis that p67^phox^ and Rac bind directly to gp91^phox^ and activates NADPH oxidase by inducing conformational changes in its flavoprotein domain [7]. Using a similar approach, different domains of p22^phox^ protein have been delineated and shown to be involved in the maturation of the gp91^phox^ protein before the assembly of the gp91^phox^/p22^phox^ heterodimer [8].

Among these approaches, an internal domain in the N-terminal part of p22^phox^ is involved in the flavocytochrome assembly, whereas the proline-rich region (PRR motif) in the C-terminal portion is responsible for NADPH oxidase activity via the binding to the p47^phox^ SH3 domain [1].

Chronic granulomatous disease (CGD) is a rare immunodeficiency disease caused by mutations in genes encoding one of the main constituents of NADPH oxidase [9]. Mutations in the *CYBB* gene encoding gp91^phox^ represent almost 60% of CGD cases. These defects largely result in a lack of protein expression (X° CGD) or, in less than 10% of CGD cases, in a decrease or a loss of oxidase activity while protein is present (X^−^ or X^+^ CGD). Recently, gene therapy has been tested in animal models and in clinical trials to attempt to reconstitute NADPH oxidase activity in X-linked CGD mice or in X-linked CGD patients [10]. Although promising results have been reported, this method still employs a retrovirus that may deliver the corrective gene into the patient's genome in locations which affect essential genes such as those involved in cancer.

Beside gene therapy, recent progress has been made with methods for the delivery of functional proteins which are based on the direct delivery of active therapeutic proteins into targeted living cells or, in the case of monoclonal antibodies, for the stimulation of specific immune responses [11]. Different strategies are used for the delivery of functional proteins such as microinjection, electroporation, liposomes or by fusion to a protein transduction domain (PTD). Among these delivery systems, liposomes represent a promising technology for the delivery of macromolecules into cells for the following reasons: (1) they are non-cytotoxic; (2), they can deliver and specifically target a large set of bioactive molecules (such as proteins, DNA or ribozymes); (3), they can protect molecules from degradation; and (4), their composition is easily modifiable. Different studies have used liposomes for the delivery of proteins such as antigens or toxins, drugs and nucleic molecules, but none of them has attempted to deliver membrane proteins.

The production of recombinant membrane proteins by the classical overexpression systems still presents a technical challenge. Among these challenges are yield, correct folding, solubility and protein integration into the lipid membrane to constitute functional proteoliposomes [12]. However, due to their hydrophobic nature, it is difficult to make membrane proteins in a native conformation for functional and structural studies in these *in vivo* systems. An interesting and attractive alternative for producing membrane proteins is the use of cell-free transcription/translation systems. These cell-free protein synthesis systems are essentially derived from rabbit reticulocytes, *Escherichia coli* lysates, or wheat germ [13]. One of the advantages of these *in vitro* expression systems is their capability to synthesize cytotoxic membrane proteins, or regulatory or unstable proteins that cannot be expressed in living organisms. The efficient expression of integral membrane proteins from prokaryotic sources has recently been demonstrated by using optimized *E. coli* cell-free systems [14]. The modification of the reaction conditions by adding chaperones, detergents or *E. coli* lipids have improved the synthesis and solubility of the expressed membrane proteins. Furthermore, solubilization of precipitated membrane proteins from cell-free expression systems and the integration into synthetic lipid vesicles result in the production of functional proteoliposomes [15]. A similar approach has been attempted in the production of functional mammalian membrane proteins, G protein coupled receptors (GPCRs) [16]. Three human GPCRs were synthesized by an *E. coli* cell-free expression system as fusion proteins with a thioredoxin tag. After integration into phospholipid vesicles, these GPCRs proteins displayed ligand-binding activities. However, the formation of functional proteoliposomes containing GPCRs with this technology requires first solubilization in the presence of detergents, and then reconstitution into lipid vesicles through long-term dialysis. Even if this work represents an important advance in the synthesis of mammalian membrane proteins by cell-free expression technology, it cannot be applied as a general strategy for producing membrane proteins due to the large size of the thioredoxin tag (11.2kDa), which may impair the protein activity.

In this study, we assessed the potential of the cell-free expression system to synthesize functional truncated forms of the gp91^phox^ subunit and its capacity to directly produce proteoliposomes containing these truncated forms. We demonstrate that all the truncated forms are expressed as soluble proteins in the presence of detergents and display fully enzymatic activity. Moreover, the addition of natural lipid vesicles in the synthesis reaction results in the formation of functional proteoliposomes which can be used as vectors in the direct delivery of the membrane protein into mammalian cell lines.

## Results

### *In vitro* expression of truncated gp91^phox^

**Figure 1:**
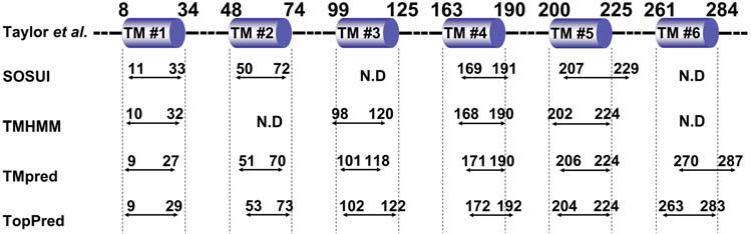
Alignment of predicted hydrophobic domains of gp91^phox^. This schema summarizes the different prediction of topology for gp91^phox^ by using different programs (Sosui, TMHMM, TMpred and TopPred) or the model published by Taylor *et al* [17].

An analysis of the prediction of the putative hydrophobic domains of gp91^phox^ protein has been carried out through the use of different programs (Sosui, TMHMM, TMpred and TopPred). An alignment of the deduced transmembrane domains from these programs with the structural model published by Taylor [17] identified 6 hydrophobic domains which differ by 1 to 10 amino acids, and 3 extracellular loops (Figure 1). Analysis of gp91^phox^ transmembrane domains by mass spectrometry has provided a more precise location of these transmembrane regions. Therefore, in this study all the generated gp91^phox^ truncated forms are based on the structural model from Taylor [17].

**Figure 2:**
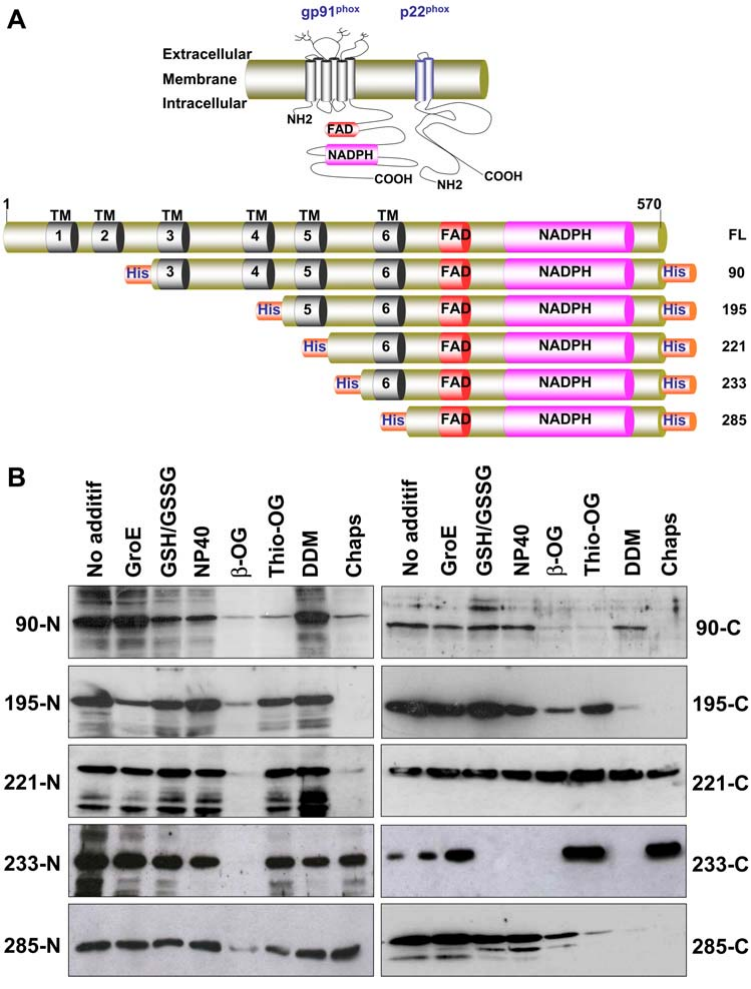
Expression of truncated gp91^phox^ proteins. (A) Schematic representation of the phagocyte NADPH oxydase and truncated forms gp91^phox^ expressed in a bacterial cell-free translation system. Gp91^phox^ full-length (FL) is presented at the top of the diagram: transmembrane domains are shown in black. FAD and NADPH binding-sites are represented in red and pink respectively. Truncated gp91^phox^ were designed with a his-tag located either at the N or at the C terminal part of the protein. (B) Analysis of the expression of the ten gp91^phox^ truncated proteins by western blot using a monoclonal antibody anti-his.

In general, the production of cytochrome *b_558_* subunits gp91^phox^ and p22^phox^ through classical over-expression systems have resulted in low yields which are incompatible for further biochemical studies [18]. Recent developments suggest that cell-free expression systems represent an alternative to classical *in vivo* expression. In order to decipher the molecular mechanisms involved in the activity of human NADPH oxidase, we generated five truncated forms of gp91^phox^. Each construct contains a histidine tag located either at the N- or at the C-terminus part of the protein (Figure 2A). These truncated gp91^phox^ proteins were first cloned into vectors dedicated to a prokaryotic expression system and then synthesized *in vitro* by using a transcription-translation system using an *E. coli* lysate.

We first set up the conditions for expressing each construct in the presence or absence of chaperones or detergents. As an initial validation, the expression of these ten truncated proteins was analyzed by western blotting using an anti-his antibody (Figure 2B). Similar results were obtained with a gp91^phox^ specific antibody (data not shown). Interestingly, the expression pattern of each construct depends on different parameters. Using the standard conditions, all the truncated gp91^phox^ proteins are expressed but at variable levels, as detected by western blotting (Figure 2B). The position of the histidine tag may have a critical effect by decreasing (e.g. gp91^phox^ 90-C) or by inhibiting (e.g. gp91^phox^ 233-C) the expression of the truncated gp91^phox^ proteins (Figure 2B). Our results suggested that the N-terminal hexa-histidine tag improves the translation efficiency either by stabilizing the messenger or by providing a better folding state of the expressed proteins.

We next studied the effect of non-ionic (*n*-dodecyl β-D-maltoside, *n*-octyl β-D-glucopyranoside, *n*-thiooctyl β-D-glucopyranoside, Nonidet P40) and zwitterionic (Chaps) detergents on the expression of gp91^phox ^truncated forms. These detergents were selected as they are mild, relatively non-denaturing and are compatible with *in vitro* expression systems as they have no inhibitory effects on the expression of membrane proteins. They were all tested at their critical micellar concentration (CMC), except for Nonidet P40 (NP40), which was 100 times more concentrated than the usual CMC (0.05 mM). Interestingly, these detergents displayed various effects on protein expression. For example, Chaps and β-OG have a strong negative effect on the expression of the gp91^phox^ truncated forms, except for constructs gp91^phox^ 221-C with β-OG and gp91^phox^ 221-C, gp91^phox^ 233-N and 233-C, gp91^phox^ 285-N with Chaps (Figure 2B). These two compounds decreased the expression of gp91^phox^ 221-N, but were compatible with the expression of gp91^phox^ 221-C (Figure 2B). In contrast, NP40, Thio-OG and DDM enhanced the expression levels for most of the gp91^phox^ truncated forms, with a stronger effect on the constructs containing a hexa-histidine at the N-terminal part of the protein (Figure 2B). These results suggest that the addition of non-ionic detergents is compatible with the synthesis of protein in a cell-free expression system, and that this may result in an enhancement of protein synthesis.

The effect of the histidine tag position on protein synthesis was also studied in the presence or absence of detergents or various compounds. Interestingly, when the histidine tag is attached to the N-terminal part of the protein, all the gp91^phox^ truncated forms were synthesized, except in the presence of β-OG and Chaps (Figure 2B). The other compounds (GroE and GSH/GSSG) had no effect on the expression of the truncated forms, except for constructs gp91^phox^ 90-N, 195-N and 233-C (Figure 2B). These data indicate that the levels of expression of gp91^phox^ truncated forms in a cell-free system depend on both the tag position and the detergent. However, there is no clear rule to determine whether a mammalian membrane protein will be expressed or not in a high yield within this optimized cell-free expression system.

**Figure 3:**
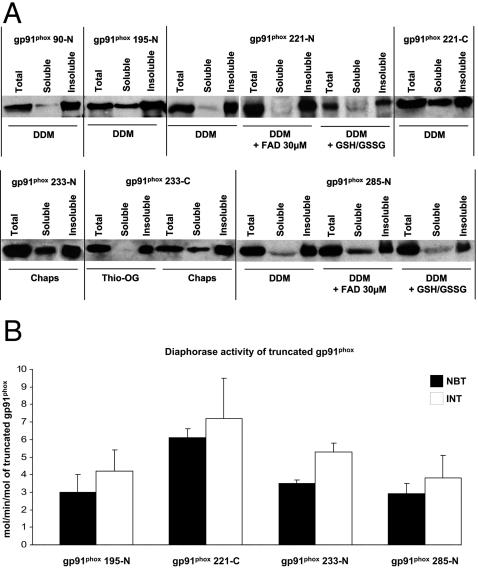
Solubility and activity of gp91^phox^ recombinant proteins. (A) Optimization of the solubility of the gp91^phox^ truncated proteins by western blot using a monoclonal antibody anti-his. (B) Diaphorase assay of purified soluble truncated gp91^phox^ proteins using NBT or INT as electron acceptor. Results were expressed as moles of NBT or INT reduced/min/mol gp91^phox^ truncated forms. Results are expressed as the average of at least two experiments±SD.

Based on these results, we sought to determine whether the truncated gp91^phox^ proteins could be expressed under a soluble form, as this form is usually considered to possess a “native-like” conformation. The solubility tests are summarized in Figure 3A. Solubilization depends on a combination of three different factors: (1) the structural properties of the membrane protein, (2) the position of the tag (gp91^phox^ 233-N vs. -C or gp91^phox^ 221-N vs. -C) and (3) the detergent by itself (DDM for gp91^phox^ 221-N/-C or Chaps for gp91^phox^ 233-N/-C). Using the standard conditions of the cell-free expression system, the gp91^phox^ truncated proteins were expressed as precipitates (data not shown). All the proteins were produced in a soluble form using DDM, except for the gp91^phox^ 233 proteins which were solubilized in the presence of Chaps. Whichever compound was added, the solubility of gp91^phox^ 90-N protein remained too low to be used for further experiments.

### Diaphorase assay of purified truncated gp91^phox^

In order to investigate the functional response of the soluble truncated gp91^phox^ proteins, it was necessary to determine whether these proteins are able to transfer electrons like the native cytochrome *b*_558_ purified from neutrophils [7]. Previous studies have shown that the glycosylation of gp91^phox^ protein is not required for its enzymatic activity [19]. However, the production of mammalian membrane protein in an *E.coli* cell-free expression system results in a non-glycosylated protein. To quantify the electron transfer from NADPH to FAD, we used two different electron acceptors: NitroBlueTetrazolium (NBT), the most commonly used electron acceptor, or IodoNitroTetrazolium (INT), which has been shown to specifically accept electrons from FAD [20]. Furthermore, the existence of a diaphorase activity in the truncated gp91^phox^ proteins would be an indicator of their correct folding status.

In order to obtain enough soluble proteins for the functional assay, we over-expressed truncated gp91^phox^ proteins in large scale reactions using the optimized conditions determined in Figure 3A (Supplementary Figure S1), followed by purification onto affinity chromatography. The soluble gp91^phox^ proteins were tested for their diaphorase activity, and for each assay 10 pmoles of purified soluble protein were used. None of the truncated proteins tested exhibited activity in the absence of cytosolic regulatory proteins (p47^phox^, p67^phox^, p40^phox^ and Rac; data not shown). By incubating the recombinant proteins with neutrophil cytosol and an optimum concentration of arachidonic acid (AA) for 10 minutes at 25°C, we were able to activate the recombinant proteins and to observe NADPH-dependent diaphorase activity.

The results for gp91^phox^ 195-N, gp91^phox^ 221-C, gp91^phox^ 233-N and gp91^phox^ 285-N proteins are represented in Figure 3B. Their specific values of activity were 3, 6.1, 3.5 and 2.9 mol/min/mol for NBT and 4.2, 7.2, 5.3 and 3.8 mol/min/mol for INT respectively. The gp91^phox^ 221-C protein showed an overall reductase activity which was higher for both NBT and INT compared to the other proteins (Figure 3B). For all the proteins, a diaphorase assay using INT as electron acceptor gave a higher turnover rate compared to the NBT values (Figure 3B). This could be explained by its higher specificity of electrons released by the FAD during the classical electron transfer pathway [21].

**Figure 4:**
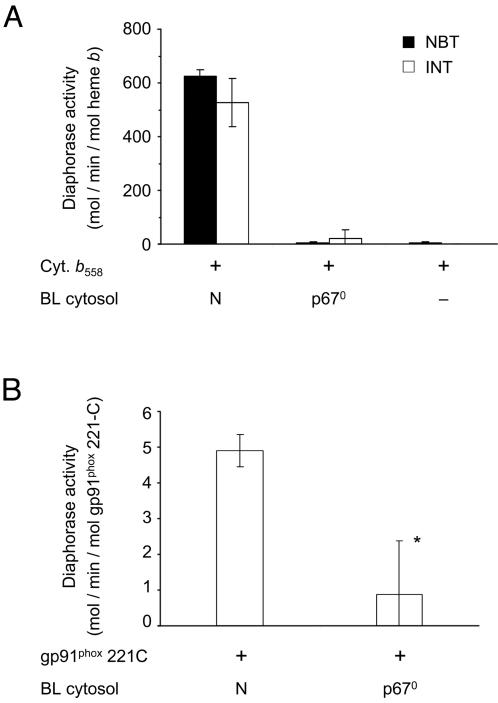
Specificity of the diaphorase activity measured with gp91^phox ^recombinant proteins. (A) Diaphorase activity measured with cytochrome *b*_558_ purified from neutrophils (10 pmol) in presence of cytosol from normal (N) or p67^phox^ deficient (p67^0^) EBV B lymphocytes using NBT (black bars) or INT (open bars) as electron acceptor. (B) Diaphorase activity measured with the gp91^phox^ 221-C protein (10 pmol) in presence of cytosol from normal (N) or p67^phox^ deficient (p67^0^) EBV B lymphocytes using INT (open bars) as electron acceptor. Results are expressed as the mean activity of three experiments±SD. * indicates results significantly different (P<0.05) from the control performed in presence of normal cytosol.

To confirm that the diaphorase activity measured with truncated gp91^phox^ proteins was directly dependent on the presence of cytosolic regulatory factors, we performed the same experiment with a cytosolic fraction isolated from control or p67^phox^-deficient EBV-B lymphocytes. First, the diaphorase activity of native cytochrome *b*_558_ purified from neutrophils was analyzed. As expected, the electron transfer was correlated to the presence of cytosolic factors p67^phox^, p47^phox^ and Rac as shown by the absence of activity measured with p67^phox^-deficient cytosol or without cytosol (Figure 4A). The diaphorase activity was in the range of 500–600 mol/min/mol heme *b*, depending on the electron acceptor used (INT or NBT). Then, the activity of the gp91^phox^ 221-C was determined after incubation with control or deficient B lymphocyte cytosol, and arachidonic acid. The diaphorase activity was in the range of 5 mol/min/mol gp91^phox^ 221-C with the control cytosol and was strongly and significantly decreased in presence of p67phox-deficient cytosol (∼0.9 mol/min/mol gp91^phox^ 221-C, Figure 4B).

Taken together, these results suggested a direct interaction between the cytosolic factors (p47^phox^, p67^phox^, p40^phox^ and Rac) and the recombinant soluble proteins, indicating an assembly of these factors and the truncated proteins as earlier described for the native cytochrome *b*_558_ during the activation of the NADPH oxidase complex [1]. Moreover, all the recombinant truncated gp91^phox^ proteins tested were soluble and showed a specific enzymatic activity, confirming that they are in a correct and active folding state.

### Expression of truncated gp91^phox^ in the presence of lipids

The acquisition of liposomes containing active membrane proteins is a useful tool in the study of their biochemical properties. Moreover, functional gp91^phox^ proteoliposomes may represent an attractive delivery system for complementing NADPH activity in the neutrophils of CGD patients.

In this study, we used natural lipids from spinach thylakoids. These are anionic lipids mainly composed of diacylglycerol derivatives. Usually, to obtain membrane protein inserted into a lipid vesicle, 2 steps are required: (1) extraction/solubilization/purification of the membrane protein from its natural source or from the over-expression system (i.e. inclusion bodies in *E.coli*) by using detergents or chaotropic agents (urea or guanidine), and (2) reconstitution into liposomes after refolding [12]. The originality of our approach results in the production of the proteoliposomes in a one-step reaction by directly adding the lipids to the reaction mixture during the synthesis.

**Figure 5:**
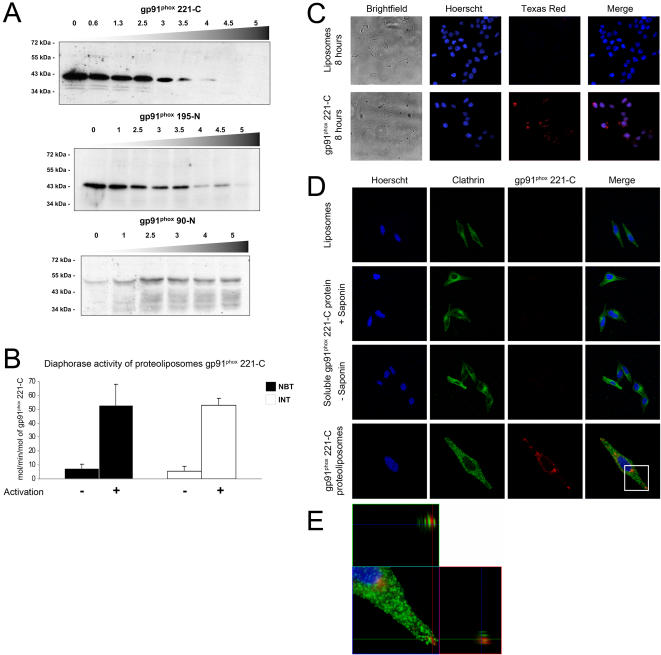
gp91^phox^ 221-C proteoliposomes. (A) Expression of membrane protein in presence of lipids analyzed by western blot using a monoclonal antibody anti-his. (B) Diaphorase assay of purified proteoliposomes gp91^phox^ 221-C using NBT or INT as electron acceptor, n≥2. (C) Delivery gp91^phox^ 221-C with proteoliposomes in HCT116 cells after 8 hours of incubation. As a negative control, cells were incubated with the same amount (0.5 µg) of empty liposomes. The proteins were detected by fluorescence microscopy using a monoclonal anti-his antibody. (D) Confocal analysis of the distribution of gp91^phox^ 221-C protein delivered with proteoliposomes in HeLa cells after 8 hours of incubation. As a negative control, cells were incubated with 0.5 µg of empty liposomes or 0.5 µg of soluble gp91^phox^ 221-C. Cells were labelled for the exogenous protein using an anti-gp91^phox^ antibody (Red) and for the endogenous Clathrin (Green). The cells were examined under a Zeiss LSM510 (NLO) laser confocal microscope. The image plane was chosen to be near the equator of the cell body and nucleus. (E) Higher magnification of the white boxed area in (D) with the respective *xz* projections.

Firstly, our goal was to determine whether truncated gp91^phox^ proteins could be expressed and integrated into natural lipids. We tested the expression of three membrane proteins, gp91^phox^ 221-C, gp91^phox^ 195-N and gp91^phox^ 90-N containing respectively 1, 2 and 4 transmembrane domains in the presence of thylakoid lipids. For the gp91^phox^ 221-C and gp91^phox^ 195-N proteins, a modulation of the expression depending on the amount of lipids added during the synthesis was observed. This effect is negligible in the presence of low concentrations of lipids (≤2.5 mg/ml), but an inhibition of the expression can be observed in the presence of higher concentrations of lipids (≥4 mg/ml, Figure 5A). Interestingly, the expression of gp91^phox^ 90-N protein in the same conditions showed the opposite effect (Figure 5A). The addition of lipids (up to 2.5 mg/ml) enhanced its expression, and higher concentrations (>2.5 mg/ml) resulted in a slight inhibition compared to 2.5 mg/ml of lipids (Figure 5A). Based on these results, we next performed large-scale expression reactions for the construct gp91^phox^ 221-C in the presence of 2.5 mg/ml of lipids. These proteoliposomes can be quickly and easily purified by sucrose gradient centrifugation, resulting in a purified protein which is largely integrated into the lipids vesicles (data not shown).

### Diaphorase assay of gp91^phox^ 221-C proteoliposomes

In order to evaluate the effect of the integration into natural lipids on the capacity to transfer electrons of the gp91^phox^ 221-C proteins, we used purified recombinant proteoliposomes containing gp91^phox^ 221-C protein and assessed its reductase activity as described for the soluble gp91^phox^ 221-C protein (Figure 3B).

Interestingly, truncated gp91^phox^ 221-C proteins integrated into natural lipid vesicles exhibit NADPH- and FAD-dependent activity that did not require the addition of cytosolic factors and AA (Figure 5B, without activation). As already described by Koshkin [22], [23], the phospholipid environment of relipidated gp91^phox^ protein has a critical influence on its folding status and on its affinity to bind co-factors such as FAD, leading to a self-active gp91^phox^ protein that only requires NADPH and FAD. This basal activity of gp91^phox^ 221-C protein in the presence of the 2 co-factors was 7.8 mol of NBT reduced/min/mol of gp91^phox^ 221-C proteoliposomes and 5.5 mol of INT reduced/min/mol of gp91^phox^ 221-C proteoliposomes. These values were comparable to those obtained for the gp91^phox^ 221-C protein expressed in the presence of detergent and activated by cytosolic factors and AA (6.1 and 7.2 mol/min/mol for NBT and INT respectively, Figure 3B). This means that the gp91^phox^ 221-C protein embedded into natural lipids is folded in a more active conformation in comparison to the detergent-soluble protein.

When 10 pmoles of recombinant gp91^phox^ 221-C proteoliposomes were incubated for 10 minutes with neutrophil cytosol (containing p47^phox^, p67^phox^, p40^phox^ and Rac) and AA at 25°C, reductase activity was enhanced by a seven fold increase for NBT and ten fold for INT (53 mol/min/mol for both NBT and INT, Figure 5B activated). Taken together, these results confirmed that the recombinant truncated gp91^phox^ 221-C protein was able to display a partial diaphorase activity when embedded into natural lipids and an increased activity after activation by cytosolic partners and AA.

### Transduction of active proteoliposomes gp91^phox^ 221-C into living cells

To test the capacity of the recombinant proteoliposomes to deliver active gp91^phox^ 221-C protein, we used a human carcinoma cell line HCT116 which is negative for gp91^phox^ messenger and proteins (data not shown).

The cells were incubated with gp91^phox^ 221-C proteoliposomes for 8 hours. The uptake of exogenous gp91^phox^ 221-C protein by the cells was detected by imunocytochemistry staining using an anti-his antibody (Figure 5C). Only cells incubated with proteoliposomes gp91^phox^ 221-C were positive for imunocytochemistry staining with a punctuated signal surrounding the cells (Figure 5C).

To confirm the cellular integration of the exogenous gp91^phox^ 221-C protein into the plasma membrane a set of experiments has been performed using freshly produced gp91^phox^ 221-C proteoliposomes. HeLa cells were incubated for 8 hours either with gp91^phox^ 221-C proteoliposomes or empty liposomes or soluble gp91^phox^ 221-C. The subcellular localization of the exogenous gp91^phox^ 221-C protein in the cells was detected by imunocytochemistry staining using an anti-gp91^phox^ antibody (54.1, in red) and an anti-Clathrin light chain subunits (sc-28276, in green). After fixation, cells were analyzed using confocal microscopy (Figure 5D). Cells incubated with the soluble protein were permeabilized or not with saponin to check for possible external binding. A red punctuated signal can be observed only with cells incubated with gp91^phox^ 221-C proteoliposomes illustrating that our results are reproducible and that the delivery is specifically due to the liposome carrier (Figure 5 C and 5D). Furthermore, the orthogonal sections show that the exogenous gp91^phox^ 221-C protein is localized into the same z sections as the clathrin clearly indicating that the protein is integrated into the plasma membrane (Figure 5D, [24]).

The use of liposomes as a delivery tool allowed the release of active membrane protein directly into the plasma membrane of cells.

### Diaphorase assay of gp91^phox^ 90-N proteoliposomes

In order to improve the system of production of mammalian membrane proteins and to further explore the effects of the gp91^phox^ protein, we produced the gp91^phox^ 90-N protein which contains 4 transmembrane domains in the presence of natural liposomes. As previously found for gp91^phox^ 90-N (Figure 5A), the optimal amount of lipids required for its synthesis was 2.5mg/ml.

**Figure 6:**
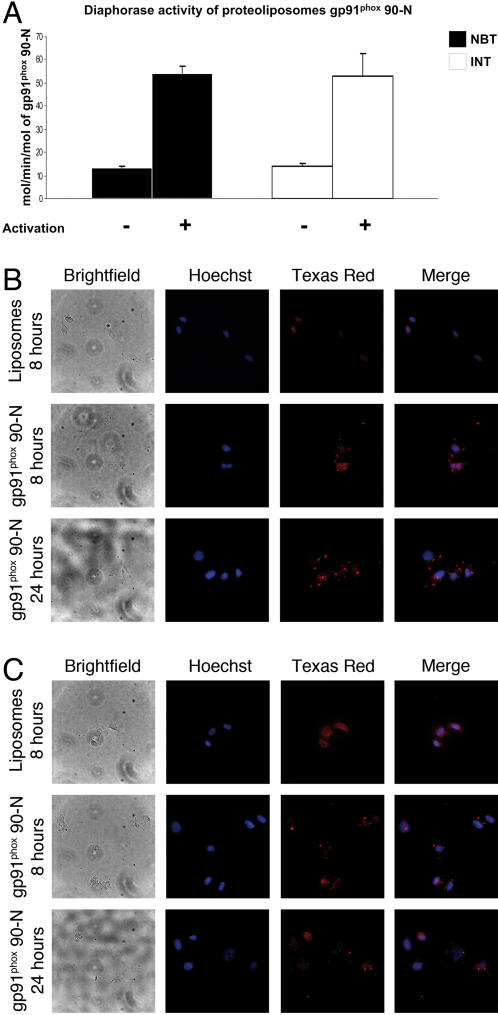
gp91^phox^ 90-N proteoliposomes. (A) Diaphorase assay of purified proteoliposomes gp91phox 90-N using NBT or INT as electron acceptor, n≥2. Cellular uptake of gp91^phox^ 90-N proteins delivered with proteoliposomes in HeLa cells after 8 or 24 hours of incubation. The proteins were detected by fluorescence microscopy using a monoclonal anti-his antibody (B) or a monoclonal anti-gp91^phox^ antibody (C).

Interestingly, as for the gp91^phox^ 221-C proteins, the gp91^phox^ 90-N proteins embedded into natural lipid exhibited an intrinsic activity without cytosolic factors and AA (Figure 6A, without activation, 13.5 mol/min/mol for NBT reduction and 14 mol/min/mol for INT reduction). These values were slightly higher than for the gp91^phox^ 221-C proteoliposomes (Figure 5B), but the addition of cytosolic extract and AA enhanced the activity of recombinant gp91^phox^ 90-N to the similar values of gp91phox 221-C proteoliposomes, indicating a fully active enzyme (54 and 53 mol/min/mol for NBT and INT respectively, Figure 6A). Embedded into natural lipids, recombinant truncated gp91^phox^ proteins displayed basal activity and a maximum turnover upon activation (Figures 5B and 6A).

### Transduction of active proteoliposomes gp91^phox^ 90-N into living cells

In order to examine the delivery of active mammalian membrane protein using liposomes as carriers, we used a human carcinoma cell line HeLa which is negative for gp91^phox^ proteins (data not shown) so as to exclude cellular specificity of the delivery.

To improve this delivery system and to follow the destiny of exogenous protein, we observed the subcellular distribution of transduced gp91^phox^ 90-N protein in HeLa cells after 8 and 24 hours by immunocytochemistry using either an anti-his antibody (Figure 6B) or an anti-gp91^phox^ antibody (Figure 6C). Interestingly, the cells incubated with gp91^phox^ 90-N proteoliposomes were positive for immunocytochemistry staining with both antibodies (Figures 6B and 6C). The higher background for the anti-gp91^phox^ antibody (Figure 6C) could be explained by its cross-reactivity with GRP 58 protein [25]. After 8 hours, the punctuated signal surrounding the cells indicated a plasma membrane localization (Figures 6B and 6C) for the delivered gp91^phox^ 90-N proteins and after 24 hours, the proteins remained detectable and localized to the plasma membrane of the cells (Figures 6B and 6C).

The delivery of recombinant mammalian membrane proteins into living cells using liposomes as a vector appears to be a powerful system in the targeting of deficient cells (Figures 5C, 5D, 6B and 6C).

## Discussion

The study of functional membrane proteins remains a challenging task, regardless of the fact that important progress has recently been made in the isolation of eukaryotic membrane proteins from natural sources or the production of membrane proteins in heterologous expression systems [26]. The failure to produce recombinant eukaryotic membrane proteins is mainly due to a misfolding and rapid degradation of the over-expressed membrane proteins. Moreover, low yields and cellular toxicity can account for the difficulty in producing membrane proteins. Recent studies on the synthesis of mammalian membrane proteins in different optimized expression systems have been explored in order to produce large amounts of recombinant proteins for functional and structural studies [27]–[30]. However, expression in these systems often results in the targeting of the membrane proteins to the inclusion bodies or to the membrane of the host cells, thus rendering their subsequent study difficult. Cell-free expression systems represent an excellent alternative to the classical *in vivo* over-expression systems [13]. These have already been used for the production of membrane proteins from *E. coli* [14], [31], [32] or from eukaryotic sources [15], [16].

The gp91^phox^ protein is a transmembrane glycosylated hemoprotein. It is the redox-subunit of cytochrome *b_558_* and the catalytic core of the phagocyte NADPH oxidase. The lack of gp91^phox^ causes an X-linked chronic granulomatous disease (CGD), a rare genetic disorder characterized by severe and recurrent infections due to the failure of O_2_^−^-generating NADPH oxidase and the absence of reactive oxygen species. Different approaches have been used to study the NADPH oxidase complex, including monoclonal antibodies [17], [33], isolation and purification of the complex from neutrophils [7], [34] and the expression of deletion mutants of the gp91^phox^ protein [6], [35] or of the p22^phox^ protein [8]. These methods have provided vital information about the regulation of this enzymatic complex through the identification of new regulatory proteins [34], the description of domains of interaction between its two subunits [8], the modification of the conformation of gp91^phox^ [33] and topological information regarding the components of the NADPH complex [17].

In this study we have developed a new method for producing fully active recombinant truncated gp91^phox^ proteins containing up to 4 transmembrane domains (Figures 2A and 2B). We show that recombinant gp91^phox^ proteins are solubilized by non-ionic or zwitterionic detergents and that they exhibit reductase activities in the absence of p22^phox^ protein. Recent studies reported that the C-terminal part of the protein (aa 221 to 570) is responsible for electron transfer from NADPH to FAD [6], [36]. However, stimulation of the reductase activities of these constructs appears to depend either on the activation domain of p67^phox^ and Rac or on the carboxyl-terminal truncated p67^phox^ fused with Rac [5]. Our finding shows that soluble gp91^phox^ 221-C displays an NADPH- and a p67^phox^-dependent diaphorase activity (Figure 3B and 4B) identical to that described by Nisimoto using truncated forms of gp91^phox^ over-expressed as inclusion bodies in *E.coli* [6]. These data confirm the specific role of the cytosolic regulatory *phox* factors in the electron transfer activation in the gp91^phox^ truncated form and led us to conclude that our optimized cell-free expression system represents a new alternative to obtaining active truncated gp91^phox^ recombinant derivatives.

Our recombinant model also supports previous works reporting that N-glycosylation on three Asn residues [Asn132, Asn149 and Asn240; 4] of the wild type gp91^phox^ protein are not required for the production of superoxide anions [19]. Although we cannot exclude the possibility that glycosylation may be involved in cytochrome *b_558_* stability in neutrophils, all the recombinant proteins produced by the cell-free expression system are not glycosylated, but display diaphorase activities.

Taken together, these results demonstrate that our optimized cell-free expression system represents a new alternative for the generation of active truncated gp91^phox^ proteins. These derivatives could be used as molecular probes to further investigate the interaction between gp91^phox^ and p22^phox^ and the subsequent conformation states of cytochrome *b_558_* at rest and upon activation of NADPH oxidase. Furthermore, this original recombinant approach will be used to study the recently discovered isoforms of gp91^phox^, mainly Nox1 and Nox4 of the Nox family [37].

Previous studies of Koshkin and Pick showed an intrinsic activity of the purified cytochrome *b_558_* from neutrophils after integration into lipids without the requirement of any activation [22], [23], implying that the lipid environment plays a crucial role in the activity and the conformation of proteins. An interesting feature of the cell-free expression system is its compatibility with natural lipid vesicles. We have demonstrated that the addition of liposomes into the reaction mixture allows direct formation of active proteoliposomes containing truncated gp91^phox^ protein or membrane proteins from various origins (Liguori *et al*., submitted). We showed that gp91^phox^ 221-C proteoliposomes display similar diaphorase activity as the soluble protein, even in absence of cytosolic factors or anionic amphiphile (7.8 mol/min/mol for gp91^phox^ 221-C proteoliposomes and 6.1 mol/min/mol for soluble gp91^phox^ 221-C). Moreover, the diaphorase activity of gp91^phox^ 221-C proteoliposomes can be enhanced 7 to 10 fold by the addition of cytosolic partners and AA, leading to a better activation of the protein. Furthermore, similar results have been obtained with gp91^phox^ 90-N proteoliposomes. These results suggest a “native-like” conformation for these gp91^phox^ constructs that was directly dependent on the lipid environment. This, to our knowledge, is the first demonstration of an active recombinant gp91^phox^ protein with four transmembrane domains directly integrated into lipids in the absence of the p22^phox^ protein.

Recently, a fully active NADPH oxidase complex was isolated onto an affinity matrix and in the absence of lipids. The constitutive activity of the isolated complex suggested a change of cytochrome *b_558_* conformation from an inactive to an active state [7]. Moreover, prenylated p67^phox^-rac1 chimera were shown to activate membrane cytochrome *b_558_ in vitro* in the absence of lipids or amphiphil reagent [38]. However, our findings point to the development of a recombinant model of neutrophil-like NADPH oxidase which allows the direct production of active and “native-like” membrane gp91^phox^ that is qualitatively similar to that of purified protein from neutrophils. Further structural data will shed light on the mechanisms of diaphorase activation of recombinant gp91^phox^ constructs in the absence or presence of lipids.

Data presented here provide conclusive results on the capacity of the optimized cell-free expression system to produce fully active proteoliposomes in a one-step reaction. Liposomes are now widely used for the delivery of various therapeutic molecules such as antibodies [11], nucleic acids [39], peptides [40] and antifungal [41] or anticancer drugs [42]. These molecules can be located either in the aqueous compartment, if soluble, or embedded into the bilayer for hydrophobic compounds. Moreover, various liposomal vesicles containing pharmaceutical agents have been developed to sustain their therapeutic actions *in vivo* by a prolonged half-life circulation, a reduced cytotoxicity and an improved cellular and tissue targeting [43].

One of the current challenges in therapy is to develop new strategies in the efficient treatment of disease. Encouraging results have recently been obtained using gene therapy to treat CGD [10]. However, even if gene therapy appears to be a promising method of treatment of rare diseases, its efficiency needs to be improved, and a number of major problems inherent to this technology remain to be solved, such as immunogenic responses against the vector, low specificity of cells expressing the protein and the random insertion of DNA into the genome of cells [44]. Therefore, an alternative or a complementary approach to gene therapy needs to be developed.

In this paper, we report the development of a new method to produce recombinant proteoliposomes containing membrane protein in a one-step reaction, and we demonstrate that these proteoliposomes can be used as carriers for delivering membrane proteins. We evaluate the ability of proteoliposomes to exhibit diaphorase activity in *in vitro* assays (Figures 5B and 6A) and to efficiently deliver truncated gp91^phox^ protein into cells targeting their plasma membrane (Figures 5C, 5D, 6B and 6C). Therefore, we demonstrated the “proof of concept” that the active embedded protein can be directly delivered to the plasma membrane of different cell lines, and we demonstrated that our proteoliposomes may represent an original approach for protein therapy. However, production by the cell-free expression system of a fully functional cytochrome *b*_558 _containing two hemes remains a challenge. The incorporation of hemin in the cell-free expression medium was first tested to evaluate the feasibility to express heme-bound protein with this expression system. Preliminary results indicate that concentrations of hemin below 1.6 µM do not interfere with the transcription/translation process suggesting that the cell-free expression system could be useful to produce recombinant cytochrome *b*_558_.

## Materials and Methods

### Materials

All the chemicals were from Sigma-Aldrich and the detergents from Calbiochem. Medium for cell culture were from Invitrogen-Gibco.

### Truncated gp91^phox^

The different versions of gp91^phox^ were obtained by PCR. The forward primers (^5′^GGAATTC**CATATG**GTTCGAAGACAACTGGACAGG^3′^ for gp91^phox^ 90, ^5′^GGAATTC**CATATG**AAAACCATCCGGAGGTCTTAC^3′^ for gp91^phox^ 195, ^5′^GGAATTC**CATATG**ATCCATGGAGCTGAACGAA^3′^ for gp91^phox^ 221, ^5′^GGAATTC**CATATG**GCAGAGAGTTTGGCTGTG^3′^ for gp91^phox^ 233 and ^5′^GGAATTC**CATATG**TTTTGGCGATCTCAACAGA^3′^ for gp91^phox^ 285) were designed to introduce a NdeI site (shown in boldface). The reverse primers (^5′^GCGTTA**CTCGAG**TCATGGAAGAGACAAGTTAGAAG^3′^ for the N-terminal position of the his-tag or ^5′^GCGTTA**CTCGAG**GAAGTTTTCCTTGTTGAAAATG^3′^ for the C-terminal position of the his-tag) were designed to introduce a XhoI site (shown in boldface) and a stop codon for the N-terminal position of the his-tag (underlined). The truncated gp91^phox^ were directly cloned in pIVEX 2.3MCS and in pIVEX 2.4NdeI vectors (Roche Applied Science).

### Cell-free expression of gp91^phox^

Expression test for each protein was performed using the RTS™ HY100 (Roche Applied Science) according to the manufacturer's instructions. We tested different compounds to enhance the expression under soluble form (i.e. “native-like” conformation): GroE chaperone, GSH/GSSG (0.01 mM/0.4 mM), Nonidet P40 (NP40, 5 mM), β-OG (*n*-Octyl-β-D-glucopyranoside, 25 mM), Thio-OG (*n*-Octyl-β-D-thioglycopyranoside, 9 mM), DDM (*n*-Dodecyl-β-D-maltoside, 0,1 mM), CHAPS (3-[(3-cholamidopropyl)-dimethylammonio]propanesulfonate, 10 mM) or liposomes at different concentration (from 1 mg/ml up to 5 mg/ml). Liposomes are obtained by evaporation of CHCl_3_/MeOH from the lipid preparation (SpeedVac System, Thermo Savant) and resuspension of the lipids in DEPC water. Sonication (3 times for 1 minute on ice, Branson Sonic power, Smithkline Company Brentford, Middlesex, UK) and filtration through a 0.22 µm filter leads to liposomes constitution. The reactions were performed during 15 h at 30°C with 180rpm shaking in the Proteomaster™ (Roche Applied Science). The solubility was assayed by centrifugation (30 minutes, 21400 g, 4°C) using a Sigma 2K15 centrifuge. The supernatant was considered as the soluble fraction (except when expressed with liposomes) and the solubility was evaluated by densitometry analysis of the western blot using ImageJ programme (NIH, USA). For scale-up experiments, RTS™ HY500 ProteoMaster from Roche Applied Science was used during 48 hours at 20°C with 990rpm shaking.

### Purification of the soluble proteins by IMAC

The soluble fraction of the reaction was equilibrated by the addition of 4 column volumes (CV) of buffer A (Na-phosphate 50 mM pH 7+500 mM NaCl+Glycerol 15%+DDM 0,1 mM+Complet EDTA free) and then loaded, at least 10 times, onto a column containing 500 µl of Talon™ resin (BD Biosciences Clonetech), pre-equilibrated with 20 CV of buffer A. The column was then washed extensively with 20 CV of buffer A+10 mM imidazol. The protein was eluted with 4 CV of buffer A+250 mM imidazol.

### Proteoliposomes purification

After production, the mixtures are centrifuged at 13000 g, 20 min at 4°C, the supernatant discarded and the pellet resuspended in 1 ml Tris 50 mM, pH 7.2. The resuspended pellet was loaded on a 3 steps discontinuous sucrose gradient (60%, 25% and 10%) prepared in Tris 50 mM, pH 7.2. The sample is loaded between 60% layer (6 ml) and 25% layer (6 ml). 2 ml of 10% sucrose fulfils the gradient. After ultracentrifugation (1 hour at 200000g at 4°C), fractions of 1 ml were collected until the bottom of the gradient and analyzed by western blotting and silver staining.

### Lymphoid Cell Line and Neutrophils

Citrate-sterile venous blood was drawn from either healthy patients or a CGD patient with previously characterized CGD AR p67^0^ after informed consent. Neutrophils and B lymphocytes were isolated by Ficoll-Hypaque density gradient centrifugation [7]. Lymphocytes were collected at the Ficoll surface and infected with the B95-8 strain of Epstein-Barr virus (EBV) as described previously [7]. The EBV-B lymphocyte cell lines were kept in culture using RPMI 1640 supplemented with 10% fetal calf serum, 2 mM L-glutamine, and 2 µg/mL Ciflox, at 37°C under a 5% CO2 atmosphere. Neutrophils were collected in the pellet after red-cell hypotonic lysis [7]. Crude membrane and cytosol fractions from both cell types were prepared as reported previously [7].

### Cytochrome *b*_558_ purification

Cytochrome *b*_558_ was purified from the plasma membranes of 10^10^ PMA-stimulated neutrophils and relipidated with L-α-phosphatidylcholine II-S as reported [19].

### Diaphorase assay in cell-free system

Electron transfer activity was measured *in vitro* using previously described protocols [45]. Purified proteins (gp91^phox^ truncated forms or neutrophil cytochrome *b*_558_), 10 pmoles, were incubated with FAD (10 µM), MgCl_2_ (5 mM), GTPγs (40 µM), 300 µg of cytosol from either human neutrophils or EBV-B lymphocytes (control or deficient in p67^phox^), and an optimum amount of AA (20 mM in EtOH) in PBS. After incubation for 10 min at 25°C, the reaction was initiated by the addition of NADPH (15 µM) in the presence of NBT (NitroBlue Tetrazolium) or INT (IodoNitro Tetrazolium) and the reduction of these compounds was followed during 30 minutes (at 595nm for ΝΒΤ, ε_595nm_ = 12.6 mM^−1^.cm^−1^ or at 500 nm for INT, ε_500nm_ = 11 mM^−1^.cm^−1^). For the proteoliposomes, the assay was performed with or without sonication.

### Cell Culture

HCT116 cells were cultured in McCoy's 5A medium supplemented with 10% heat-inactivated fetal bovine serum. HeLa cells were cultured in D-MEM medium supplemented with 10% heat-inactivated fetal bovine serum. For immunocytochemystry experiments, cells were cultured in eight-well slide chambers (Nalgen Nunc International) and treated with purified gp91^phox^ proteoliposomes or soluble gp91^phox^ protein at final concentration of 0.5 µg per 1×10^6^ cells and incubated for 8 or 24 hours before imunocytochemistry.

### Imunocytochemistry

After incubation, cells were washed 2 times 5 minutes in PBS and fixed for 10 minutes in PFA 4% at 25°C. They were permeabilized 10 minutes in PBS, saponin 0.1% at 25°C, followed by 1 hour incubation at 25°C in blocking solution (PBS, 0.1% saponin, milk 5%) and over-night incubation at 4°C with primary monoclonal antibody (54.1 or anti-his, Euromedex clone 1DM-1H7) diluted at 1∶1000 in blocking solution. After 3 washes in PBS for 5 minutes cells are incubated with a secondary antibody in blocking solution at 1∶1000 during 1 hour at 25°C (goat anti-mouse Alexa-fluor 546, Molecular Probes). For confocal analysis, cells were incubated 5 hours at 25°C with Clathrin antibody (Clathrin LCA (H-55): sc-28276; Santa Cruz Biotechnology, Inc) diluted at 1∶500 in blocking solution, washed 3 times in PBS for 5 minutes cells and incubated with a secondary antibody in blocking solution at 1∶1000 during 1 hour at 25°C (goat anti-rabbit Alexa-fluor 488, Molecular Probes). Cells were washed 3 times 5 minute with PBS and nuclei were stained with Hoechst 33258 (1∶2000) during 5 minutes at 25°C, washed again and then mounted. Samples were analyzed with an inverted Nikon Eclipse TE2000-E equipped with epifilters for the different fluorochromes using a 60× immersion objective or under a Zeiss LSM510 (NLO) laser confocal microscope.

## Supporting Information

Figure S1 Example of solubility after scale-up production detected by Coomassie blue staining and by western blotting. The solubility of gp91^phox^ 285-N and gp91^phox^ 221-C proteins was analyzed, after scale-up synthesis, by Coomassie blue staining and by western blotting using an anti-his antibody. For the gp91^phox^ 285-N protein, 15% of the produced protein was found in the soluble fraction and for the gp91^phox^ 221-C protein, 26% of the synthesized protein was recovered in the supernatant after centrifugation. The levels of solubilization for the gp91^phox^ 195-N and gp91^phox^ 233-N proteins were comparable to those of gp91^phox^ 285-N (data not shown).
(2.31 MB TIF)
