# Supplementary figures and images for: Liposome-Mediated Cellular Delivery of Active gp91phox
Source: PLoS One. 2007 Sep 12;2(9):e856. doi: 10.1371/journal.pone.0000856 (PMC1955831; doi:10.1371/journal.pone.0000856)

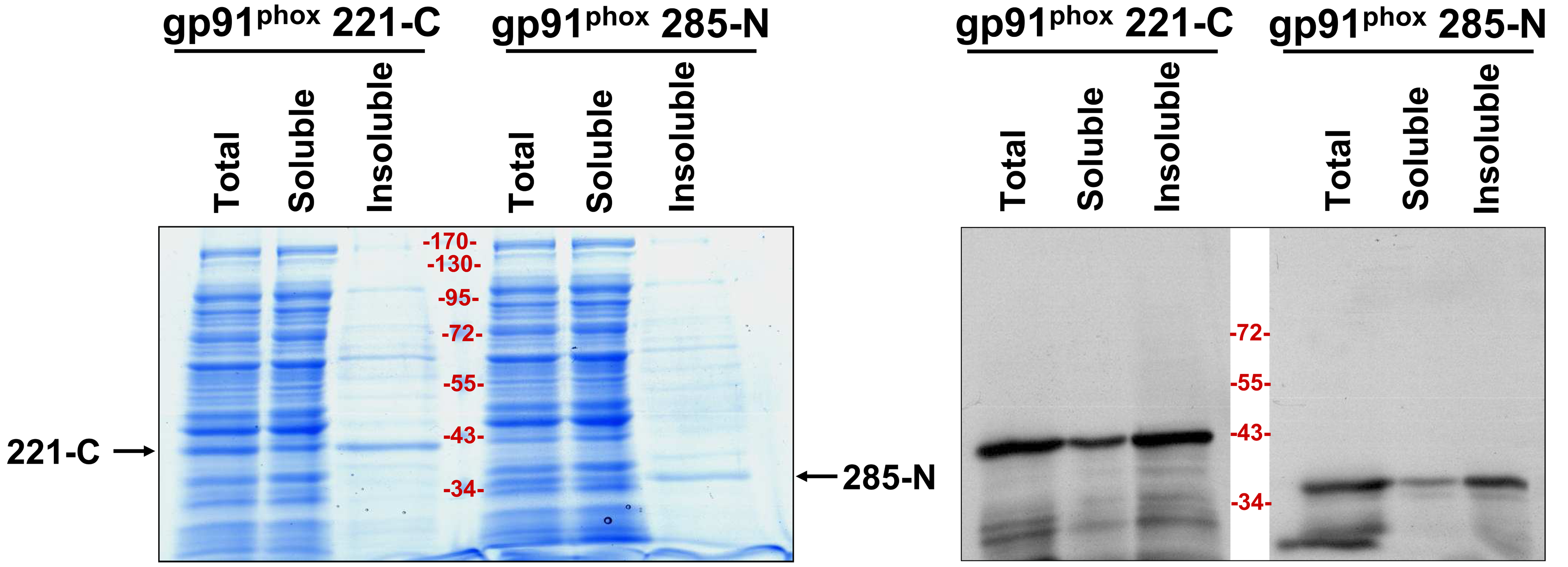

Supplement: Figure S1 — Example of solubility after scale-up production detected by Coomassie blue staining and by western blotting. The solubility of gp91phox 285-N and gp91phox 221-C proteins was analyzed, after scale-up synthesis, by Coomassie blue staining and by western blotting using an anti-his antibody. For the gp91phox 285-N protein, 15% of the produced protein was found in the soluble fraction and for the gp91phox 221-C protein, 26% of the synthesized protein was recovered in the supernatant after centrifugation. The levels of solubilization for the gp91phox 195-N and gp91phox 233-N proteins were comparable to those of gp91phox 285-N (data not shown). (2.31 MB TIF) [file pone.0000856.s001.tif]
